# Normalization of Activated Partial Thromboplastin Time Correlates with Low Levels of Dabigatran in a Patient with Severe Sepsis

**DOI:** 10.1155/2015/137504

**Published:** 2015-06-29

**Authors:** Rikke Ebenhard Højland, Stine Borch Thorup, Bodil Steen Rasmussen

**Affiliations:** Department of Anesthesia and Intensive Care, Aalborg University Hospital, Hobrovej 18-22, 9000 Aalborg, Denmark

## Abstract

The oral anticoagulant dabigatran etexilate can be a challenge when patients need acute surgery. Sepsis and acute renal failure exacerbate the anticoagulant effect. There is no specific reversal agent for dabigatran etexilate, but it can be removed by hemodialysis. We present a case where a patient treated with dabigatran etexilate was admitted to intensive care unit with severe sepsis and acute renal failure and in need of bilateral lower limp amputation due to ischemia. The patient had severe coagulopathy and was treated with continuous venovenous hemofiltration in attempt to remove dabigatran etexilate before surgery.

## 1. Introduction

Oral anticoagulant dabigatran etexilate is used for the prevention of stroke and systemic embolism in patients with atrial fibrillation, for treatment of deep venous thrombosis and pulmonary embolism. A predictable pharmacodynamic effect makes fixed dosing without routine coagulation monitoring possible but may imply risks in critically ill patients. In critically ill patients with sepsis and renal failure the anticoagulant effect of dabigatran etexilate and the risk of bleeding can be significantly increased. Additionally, patients in need of acute surgery may be a challenge as no specific reversal agent exists.

We present a case where dabigatran etexilate caused severe coagulopathy in a patient with severe sepsis and acute renal failure in need of bilateral lower leg amputation.

## 2. Case Presentation

A 75-year-old woman was admitted to hospital with sepsis. The source of infection was bilateral ischemic leg ulcers colonized with* Pseudomonas aeruginosa*. The patient had a past medical history of arteriosclerosis, chronic obstructive pulmonary disease, hypertension, stroke, and atrial fibrillation. She received antithrombotic treatment with dabigatran etexilate 220 mg/day and aspirin 75 mg/day, both stopped at admission.

Appropriate antibiotic treatment was initiated and it was decided that a bilateral lower leg amputation was indicated for source control of the infection.

However, by then the patient was suffering from severe sepsis and acute renal failure. Arterial blood gas analysis showed a compensated metabolic acidosis with base excess of −9.5 mmol/L and lactate of 2.5 mmol/L. Blood sample analysis showed creatinine of 207 *μ*mol/L, C-reactive protein of 165 mg/L, and white blood cell count of 37.6 × 10^9^/L. Coagulation was severely affected with international normalized ratio (INR) of 2.2 and activated partial thromboplastin time (aPTT) of 148 s. The platelet count was normal at 436 × 10^9^/L.

Thus the surgery was postponed due to an expected high risk of bleeding due to sepsis and the preadmission dual antithrombotic therapy. It was decided to lower the concentration of serum dabigatran by dialysis. Continuous venovenous hemofiltration (CVVH) was chosen at the intensive care unit (ICU) due to severe sepsis induced hemodynamic instability.

At the start of CVVH aPTT had increased to 183 s with a decrease to 167 s after 12 hours of CVVH. The patient's condition was improved and it was decided to continue CVVH and postpone the surgery until the coagulation parameters were acceptable.

The patient was treated with CVVH for 4 days and during that time a hemodynamic stability and normal diuresis were obtained. Creatinine clearance increased from 11 mL/min to 33 mL/min during this period. INR was normalized and moderately prolonged aPTT of 61 s was considered acceptable. The bilateral lower leg amputation was performed successfully and the perioperative bleeding was minor with a total blood loss of 585 mL.

## 3. Discussion

Dabigatran etexilate is rapidly converted into its active form dabigatran. Dabigatran is a potent and reversible direct inhibitor of thrombin both in its fibrin-bound and in its unbound form and of thrombin-induced platelet aggregation. Peak plasma concentrations are reached after approximately 2 hours after oral administration. The volume of distribution is 60–70 L. Elimination half-life time is 12–17 hours. Approximately 80% of the active drug is eliminated in the urine, and dabigatran etexilate is therefore contraindicated in patients with severe renal impairment, that is, creatinine clearance below 30 mL/min [1].

**Figure 1:**
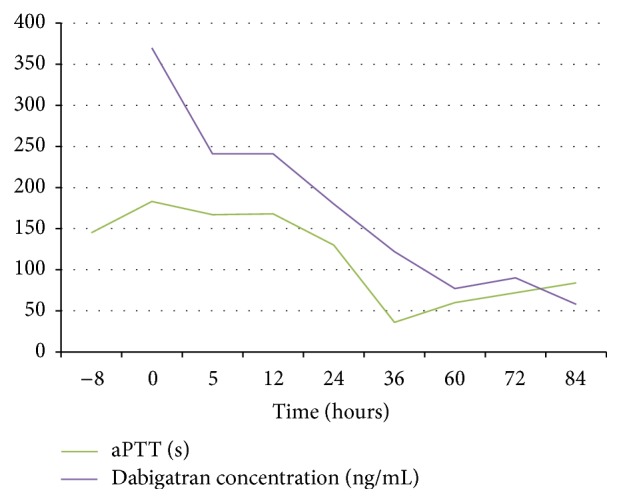
aPTT and dabigatran plasma concentration versus time after CVVH.

Ecarin clotting time and thrombin clotting time are the most sensitive tests for evaluating the anticoagulant effects of dabigatran, but these tests are often not available in the daily clinical practice. aPTT is widely available and can be useful in determining excessive anticoagulant activity but cannot be used for precise evaluation of the anticoagulant activity [2]. In our case aPTT was used to evaluate the effect of CVVH on removing dabigatran. To gain knowledge we collected numerous blood samples for measurement of the plasma concentration of dabigatran, which is not available in the acute situation at our hospital. These measurements showed that the curves for plasma dabigatran and aPTT were almost parallel, indicating that aPTT can be used to guide the elimination of dabigatran in ICU (Figure 1).

The low binding of dabigatran to plasma proteins being 35% makes it possible to remove dabigatran by hemodialysis. A 60% reduction in plasma dabigatran after 2-3 hours of hemodialysis is described [3]. However, a significant rebound effect of up to 87% has been reported in patients with acute bleeding treated with hemodialysis [4].

There is limited data on the effect of CVVH on removing dabigatran. A case report on a suicidal patient with moderate renal impairment taking an overdose of 9 grams of dabigatran etexilate showed a half-life time of 14.1 hours when treated with CVVH [5].

In our case the patient needed CVVH for 4 days before aPPT was nearly normal. During the same period the patient had preserved her own diuresis and increased creatinine clearance, which may have contributed to the elimination of dabigatran. At no point there was clinical or paraclinical suspicion of disseminated intravascular coagulation (DIC). The patient recovered hemodynamically on treatment with fluids and CVVH, which made the postponing of the operation feasible.

Postponement of the surgery minimized the additional risk of bleeding caused by both aspirin by itself and dual antithrombotic treatment.

The removal of dabigatran with CVVH appears to be significantly slower than that described with conventional hemodialysis and can therefore not be recommended for acute removal of dabigatran. Normalization of aPTT correlated with low plasma dabigatran and the present case showed that aPTT can be used as a surrogate of plasma dabigatran in septic patients with no signs of DIC.
